# Transitions to sustainable management of phosphorus in Brazilian agriculture

**DOI:** 10.1038/s41598-018-20887-z

**Published:** 2018-02-07

**Authors:** Paul J. A. Withers, Marcos Rodrigues, Amin Soltangheisi, Teotonio S. de Carvalho, Luiz R. G. Guilherme, Vinicius de M. Benites, Luciano C. Gatiboni, Djalma M. G. de Sousa, Rafael de S. Nunes, Ciro A. Rosolem, Fernando D. Andreote, Adilson de Oliveira, Edson L. M. Coutinho, Paulo S. Pavinato

**Affiliations:** 1 School of Environment, Natural Resources and Geography, Bangor University, Thoday Building, LL57 2UW Bangor, UK; 2 College of Agriculture ‘Luiz de Queiroz’, University of São Paulo - ESALQ-USP. Av. Pádua Dias, 11. CEP, 13418-900 Piracicaba, SP Brazil; 3 Federal University of Lavras - UFLA. Campus Universitário, PO Box 3037, CEP 37200-000 Lavras, MG Brazil; 4 Brazilian Agricultural Research Corporation, Embrapa Soils. Rua Jardim Botânico, 1024, CEP 22460-000 Rio de Janeiro, RJ Brazil; 5 Santa Catarina State University - UDESC. Av. Luís de Camões, 2090, CEP 88520-000 Lages, SC Brazil; 6 Brazilian Agricultural Research Corporation, Embrapa Cerrados. BR 020, Km 18 Planaltina. PO Box 08223, CEP 73310-970 Brasília, DF Brazil; 7 São Paulo State University, FCA/UNESP. Rua José Barbosa de Barros, 1780, CEP 186010-307 Botucatu, SP Brazil; 8 Brazilian Agricultural Research Corporation, Embrapa Soybean, PO Box 231, CEP 86001-970 Londrina, PR Brazil; 9 São Paulo State University, FCAV/UNESP. Via de acesso prof. Paulo Donato Castellane, s/n. km 5, CEP 14884-900 Jaboticabal, SP Brazil

## Abstract

Brazil’s large land base is important for global food security but its high dependency on inorganic phosphorus (P) fertilizer for crop production (2.2 Tg rising up to 4.6 Tg in 2050) is not a sustainable use of a critical and price-volatile resource. A new strategic analysis of current and future P demand/supply concluded that the nation’s secondary P resources which are produced annually (e.g. livestock manures, sugarcane processing residues) could potentially provide up to 20% of crop P demand by 2050 with further investment in P recovery technologies. However, the much larger legacy stores of secondary P in the soil (30 Tg in 2016 worth over $40 billion and rising to 105 Tg by 2050) could provide a more important buffer against future P scarcity or sudden P price fluctuations, and enable a transition to more sustainable P input strategies that could reduce current annual P surpluses by 65%. In the longer-term, farming systems in Brazil should be redesigned to operate profitably but more sustainably under lower soil P fertility thresholds.

## Introduction

Two main strategies exist to meet rising global food and biofuel demand: intensify the existing agricultural land area as much as possible, and/or expand into areas with native vegetation which would be detrimental to global biodiversity. A recent analysis^1^ suggested that global crop yields are not increasing sufficiently rapidly to avoid the less sustainable second option (i.e. agricultural expansion), unless more effort is focused on the first option (agricultural intensification). A key challenge for society is to achieve agricultural intensification sustainably without further depletion of natural capital, degradation of the environment, or threats to human well-being^2–4^. Brazil is one example of a nation whose agricultural output has increased rapidly in recent decades due to advances in agronomic practices (e.g. improved varieties, double cropping and no-tillage cultivation systems), investment in agrochemicals (e.g. lime and fertilizers) and expansion of the cultivated land area^5–7^ (Figs S1, S2 and supplementary discussion on the green revolution in Brazil). Important frontier areas of cropland expansion have been in Mato Grosso and Pará States, and in the Matopiba region (Maranhão, Tocantins, Piauí and Bahia states), and these areas are expected to continue expanding up to 2030 and beyond^8^.

Tollefson^9^ considered Brazil a global farm because of its large potential contribution to world food production. It is the second largest global supplier of food and agricultural products, and is forecast to be the foremost supplier to meet future global food demand^7^. The country’s enormous land base, generally favourable climate and deep soils provide large potential to further expand agricultural output through both agricultural intensification and controlled cropland expansion. For example, an increase in both crop yield and cropping area is likely needed to meet future sugar and bioethanol demand^10^. The intensification of beef production (e.g. through higher stocking rates, improved grass varieties and better grassland management) could release existing low-input, degraded pastureland for conversion to intensive cropland without further disturbance to natural ecosystems^11,12^. According to Sparovek *et al*.^12^, 469 Mha of native vegetation (57% of Brazil’s total land area) is currently under government protection, and it remains an important national sustainability goal to preserve these areas^13^. However, this still leaves 114 Mha of native vegetation potentially exploitable for cropland expansion within the current government limits on deforestation^12^. The considerable importance of agriculture to Brazil’s economy, and the large contribution Brazil’s agriculture makes to global trade as an exporter of soybean (*Glycine max*) and meat, reinforce the need to merge its future agricultural intensification with sustainable use of natural resources and limited environmental impact.

A major economic and environmental consideration in expanding Brazilian agriculture is the increased fertilizer requirement for higher agricultural output, and in particular phosphorus (P). Since most Brazilian soils are highly P fixing, large inputs of P fertilizers over and above crop P offtake are regularly applied to overcome the rapid immobilization of inorganic P that occurs in highly weathered soils rich in iron (Fe) and aluminium (Al)^14,15^. Expansion of Brazilian cropland by either converting degraded low-input pasture or native savanna will therefore require considerable amounts of P fertilizer^5,14,16^. Currently, Brazil’s phosphate rock (PR) mines supply only limited amounts of P due to the igneous rock’s low P solubility and high processing costs, and about 60% of inorganic P fertilizer used in Brazilian agriculture is imported^17^. More recent geological prospecting has identified several other PR reserves in Brazil, including those of sedimentary origin with higher P solubility^18^. However, logistical, environmental and tax issues are preventing these reserves from being explored in the short term. With increasing geopolitical concerns over future global PR accessibility and price volatility, a continued reliance on P imports may considerably increase the costs of food production in Brazil^19,20^. The high demand for fertilizer P, and heavy reliance on imports, makes Brazilian agriculture particularly vulnerable to future P scarcity, or sudden fluctuations in the cost of P; for example, such as occurred in 2008 when the price of PR rose by 800% over a 12–18 month period^19^. It is also widely recognized that global PR reserves are a finite and critical natural resource^21,22^, and that unused P is a costly pollutant of inland and coastal waters^23,24^. Alternative management strategies to reduce reliance on imported P, and use P more efficiently, will therefore become increasingly important if Brazilian farming systems are to be sustainable well into the future.

Here we examine the current and future P demand of Brazilian crop production, and investigate transitional strategies for meeting this demand more sustainably by reducing reliance on costly and finite PR resources. We hypothesized that there is large scope to improve the efficiency and sustainability of P use in Brazil by accounting for the potential stocks of secondary P that could substitute for P imports and increase the resilience of Brazilian agriculture to future P scarcity, or sudden P price fluctuations. We further investigated how radical any change in fertilizer P inputs up to the year 2050 needs to be to reduce Brazil’s P surplus in agriculture to near zero.

## Results

### Brazil’s phosphorus demand: past, present and future

Total annual P fertilizer use in Brazil has increased from an average of 0.04 Tg in 1960 to ca. 2.2 Tg in 2016 (Fig. S3A). This rapid rise in P fertilizer use has contributed substantially to the green revolution in Brazil, but fertilizer P inputs are twice plant demand, and have been since 1970 (Fig. S3A). The vast majority of this mineral P fertilizer is applied to cultivated crops (particularly maize (*Zea mays*), soybean and sugar cane (*Saccharum sp*)). Only about 1.5% of national P fertilizer consumption is attributed to pastureland, despite occupying substantial areas of marginal and degraded land (166 Mha)^8^. The average annual P fertilizer rate on all crops is currently ca. 25 kg P ha^−1^ yr^−1^ (Fig. S3B), but there is large regional variation. For example, while the P rate applied to soybean is around 25 kg P ha^−1^ in Paraná state (fertile soils), the average rate is 35 kg P ha^−1^ in Goiás state, and 50 kg P ha^−1^ in the Matopiba region, where a higher proportion of the soils are still responding to P fertilizer^25^. Typical annual fertilizer P rates on maize range from 35–60 kg P ha^−1 ^^25^, whilst sugarcane typically receives 50–80 kg P ha^−1^ for its establishment, and a further annual application of 10–15 kg P ha^−1^ after the third year of the usual 5, 6 or 7-year continuous growing cycle^26^.

**Figure 1:**
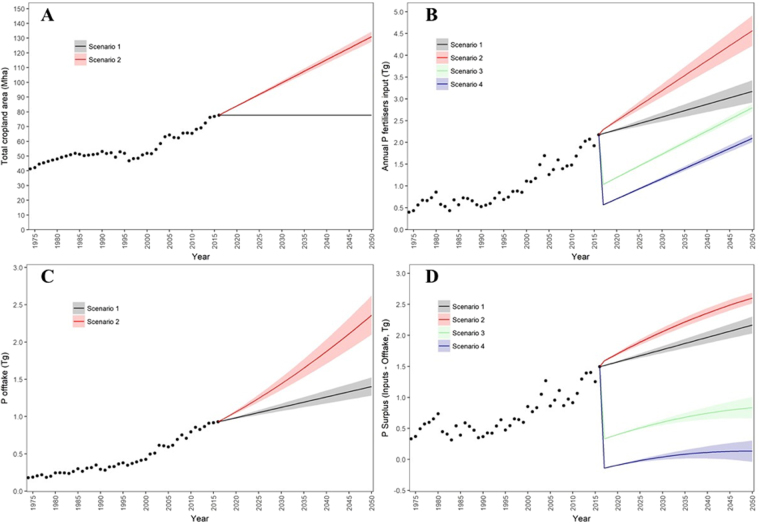
Brazil’s cropland area from 1975 to 2050, and effects of future cropland intensification (scenarios 1 and 2) and P input reduction strategies (scenarios 3 and 4) on annual P fertilizer demand (**B**), annual crop P offtake (**C**) and annual P surplus (**D**). Scenario 1 - intensify existing cropland area; Scenario 2 - intensify existing cropland area + expand cropland into native Cerrado and degraded pasture areas; Scenario 3 – ratio of P inputs:outputs in cropland areas is 1:1; Scenario 4 – ratio of P inputs:outputs in cropland areas is 0.5:1. Coloured bands give the uncertainty surrounding each scenario prediction.

The rate of increase in P fertilizer use over the last 20 years (5.5% yr^−1^) is much greater than the average rate of cropland expansion over the same period (2.6% yr^−1^), Fig. 1A,B. This largely reflects the higher starter rates of P fertilizer used to overcome P fixation in new frontier areas converted to cropland, but also includes the additional P fertilizer inputs needed for the intensification of the existing cropland, especially where farmers have adopted double cropping (typically soybean and maize). For example, the areas with double cropping have increased from 3 M ha to nearly 12 M ha in Brazil over the last 10 years^27^, and this is evident as a sharp increase in total annual crop P offtake (Fig. 1C). This increased use of fertilizer P has led to considerable surplus P accumulation in Brazil’s soils enabling increased soil P fertility. For example, the annual P surplus (calculated as total fertilizer and manure P inputs minus crop P offtake) increased nearly fivefold from ca. 0.34 Tg in 1974 to ca. 1.49 Tg in 2016 (Fig. 1D). The total P surplus in 2016 represented nearly 70% of the P fertilizer used in that year and emphasizes how inefficient current P use is in Brazil. These trends could be a significant future drain on national and global PR resources if they were to continue.

To assess the magnitude of future P fertilizer demand and potential P surpluses in Brazil up to the year 2050, we examined two cropping intensification/expansion scenarios:

### Scenario 1: Crop production will intensify only on existing land areas

Further sustainable intensification of Brazilian agriculture without increasing the cropland area could be achieved through adoption of improved agronomic practices and technologies: for example, through the zoning of areas with different climates to optimise crop varietal choice^7,9^. Annual yields of the three major cultivated crops (maize, soybean and sugarcane) have increased at a national average rate of 1.17, 2.84 and 0.6% per year, respectively over the last 20 years (Fig. S2). If sustained, these rates of intensification would increase the yields of these crops to 9.6, 4.2 and 92.4 t ha^−1^ by the year 2050, respectively. These forecast yield levels are well within the maximum yield potential of these crops considering both natural limitations (e.g. water availability)^10,28^, and data from field trials^29^. Combining the trends in yield for these three main crops gives an average rate of yield increase of 1.5% per year, which is identical to the annual increase in crop yields reported globally by Ray *et al*.^1^. Although there is large regional variation in crop yields across Brazil, we predicted that if the average yield of all annual crops continued to increase at 1.5% yr^−1^, and if P fertilizer use increased commensurately with increased yield potential (as currently recommended to farmers), then fertilizer demand could reach 3.2 Tg (41 kg P ha^−1^ yr^−1^) by 2050 leading to an annual surplus of 2.2 Tg (Fig. 1D). This demand represents an increase of 1 Tg (46%) compared to 2016 usage, and 15% of the anticipated global demand for inorganic P fertilizer on cropland (20.8 Tg) in 2050^16^.

### Scenario 2: Crop production will expand into frontier areas and intensify on existing areas

Rising global market demand for food has encouraged the recent rapid expansion of cultivated crops into native Cerrado and existing degraded pasture areas in central and North Brazil^6^. Regional variation in this rate of cropland expansion is wide. For example, maize production in Matopiba is expanding into degraded pastureland at over twice the national rate^30^. This frontier area now produces over 10% of the country’s soybean production, and is forecast to nearly double its production over the next decade^30,31^. If the current average annual rate of all cropland expansion (2.6% yr^−1^) across Brazil continues, we estimated the cropland area would increase to 131 Mha by 2050 (Fig. 1A). This represents an increase of 53 Mha in cultivated annual crops compared to 2016, which is slightly greater than the increase of 38 Mha (mainly in soybean and sugar cane) projected by the Brazilian Ministry of Agriculture^7,30^, but well within the 130 Mha (native vegetation plus degraded grassland) that is potentially suitable for conversion^12^. Conversion to cropland would require additional P fertilizer to overcome soil P fixation, termed by Roy *et al*.^14^ as a P fixation tax directly linked to soil P retention capacity^5^. For example, initial fertilizer inputs to increase P availability to critical levels in Cerrado soils can range from 26 to 122 kg P ha^−1^ for sandy to clayey soils, respectively^25^. In our analysis we assumed a conservative P fixation tax of 35 kg P ha^−1^ in the first year of cropping in addition to an average annual rate of P application of 25 kg P ha^−1^ for all new frontier cropping areas. Combining the forecast P inputs for the intensification of existing cropland (Scenario 1) together with the forecast P inputs to new frontier areas, the annual P fertilizer demand in Brazil in the year 2050 was estimated at 4.6 Tg (35 kg P ha^−1^ yr^−1^), leading to an annual surplus of 2.6 Tg (Fig. 1D). This demand represents an increase of 110% in total P consumption compared to 2016, and 22% of the anticipated global fertilizer P demand in 2050. Future fertilizer demand in Brazil does therefore constitute a significant drain on finite global P resources, requiring transitional strategies to reduce this demand and improve efficiency.

### Secondary sources of phosphorus in Brazil

A key P stewardship strategy to improve the efficiency and sustainability of P use in the food chain is to re-use (recover and/or recycle) secondary sources of P as a substitute for the imports of highly soluble inorganic fertilizers derived from PR^32,33^. This requires an understanding of the potential stocks of secondary P present in Brazil.

#### Secondary bioresources in Brazilian agriculture

Agriculture produces a number of bioresources or processing residues that could be potentially recycled back to land as secondary sources of P. Bioresources have the added advantage over mineral fertilizers in that they contain useful organic matter and water holding properties for improving general soil quality and reducing P fixation in tropical environments^34–36^.

**Figure 2:**
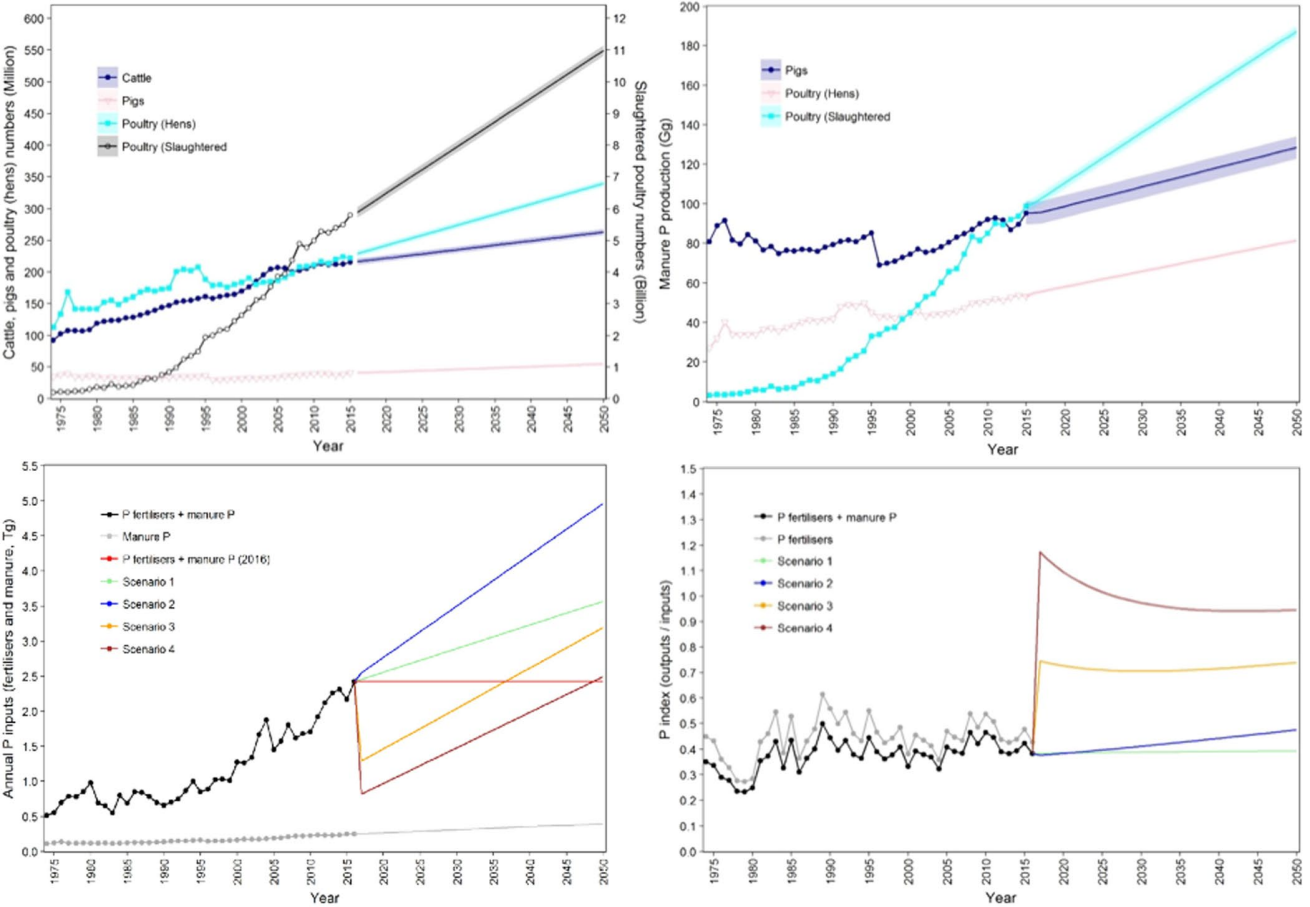
Brazil’s animal numbers (**A**) and manure P production from pigs and poultry (**B**) from 1975 to 2050, and the contribution of manures to total annual P inputs (**C**) and P efficiency index (**D**) up to 2050 and for each scenario. Scenarios are as for Fig. 1 and coloured bands give the uncertainty surrounding predictions.

Animal numbers in Brazil have increased rapidly in recent decades and generate significant amounts of manure P (Fig. 2A,B). The manure from confined cattle, pigs and poultry that is currently recycled to cropland is estimated to contain 267 Gg P (Table 1), with pigs and poultry providing the majority (93%) of this secondary P resource. Manure from unconfined cattle was not included in this analysis because it is largely dispersed within pasture areas since animals graze all year in Brazil at low (1.1 head ha^−1^) stocking densities^11^. Based on the average rate of increase in cattle, pig and poultry numbers over the last 10 years (Fig. 2B), the total manure recycled to land can be expected to provide 421 Gg P yr^−1^ by the year 2050, or 13 and 9% of Brazil’s future P demand in 2050 for scenario 1 and 2, respectively (Table 1). Amounts of biosolid P currently produced from wastewater treatment was estimated at only about 1 Gg (Table 1). According to Andreoli *et al*.^37^, only one third of the human population have combined sewage collection and treatment, and only about 15% of the treated wastewater biosolids that are produced are recycled to land. Biosolid P production from Brazil’s population is increased to only ca. 1.1 Gg in the year 2050 if current collection and treatment infrastructure remains the same, and suggests there is considerable underutilization of potential wastewater P resources in Brazil.

**Table 1:** Amounts of P contained in different secondary bioresources in Brazil annually. For 2050 predictions, confidence intervals are given in parenthesis for those based on trend analysis.

Resource type	Quantities of dry solids (DS) in 2015 (Tg)	Quantities of P in 2015 (Gg)	Quantities of dry solids (DS) in 2050 Tg	Quantities of P in 2050 (Gg)	Reference
Animal manures					
Confined cattle	3.2	19.2	4	24	^64^
Pig	40.3	95.3	54.4 (±2.4)	128.5 (±5.69)	^61^
Poultry	—	—	—	—	—
Hens	222.1	53.3	339.7 (±4.9)	81.5 (±1.18)	^62^
Slaughtered poultry	5796.2	98.8	10985.9 (±167)	187.3 (±2.85)	These authors
Total (animal manures)	—	**266.7**	—	**421.3**	—
Sugar cane	—	—	—	—	—
Filter cake	3.2	32.2	8.7	87.3	^33^
Wastewater biosolids	0.8	0.96	0.9	1.1	^32^
Total (biosolids)	—	**33.1**	—	**88.4**	—
Total (animal + biosolids)	—	**299.8**	—	**509.7**	—

The processing of sugarcane to produce sugar and ethanol produces a filter cake (30% dry matter) and a liquid effluent (vinasse) that can be beneficially recycled to land^38^. Total production of filter cake is currently estimated to be ca. 3.2 Tg of dry solids (DS) containing 32 Gg of P (Table 1). Based on the forecasted increase in sugarcane area and yields, the amount of potentially recyclable filter cake P was estimated at 87 Gg by the year 2050, or 2–3% of P demand. Liquid vinasse is more difficult to apply and contains much less P than filter cake^38^; thus it was not considered to be a significant P bioresource. As pre-harvest burning is now being phased out in Brazil, the sugarcane crop (as do other crops) produces significant amounts of straw residue (ca. 90 Tg yr^−1^) which can be generally beneficial for erosion control, nutrient cycling (e.g. contains 45 Gg of P) and improved soil C content^39^. However, this straw is more likely to be partially, or fully, removed for use as a biofuel, or for cellulosic ethanol production. Other crop residues exported off the field (e.g. coffee (Coffea) and cotton (Gossypium)) have been combined with localized sources of manures to make composts, but we considered the amounts returned to land are relatively small nationally as these crops occupy relatively small areas^27^.

Hence, we estimated that secondary bioresources currently produced on an annual basis in Brazil contain 0.3 Tg of P (Table 1), which represents 14% of current annual fertilizer P use. This secondary P resource will rise to 0.5 Tg of P in 2050, which would still only represent 16% and 11% of Brazil’s anticipated fertilizer use for scenarios 1 and 2, respectively. Alternative secondary sources of P therefore need to be explored.

#### Legacy soil P

**Figure 3:**
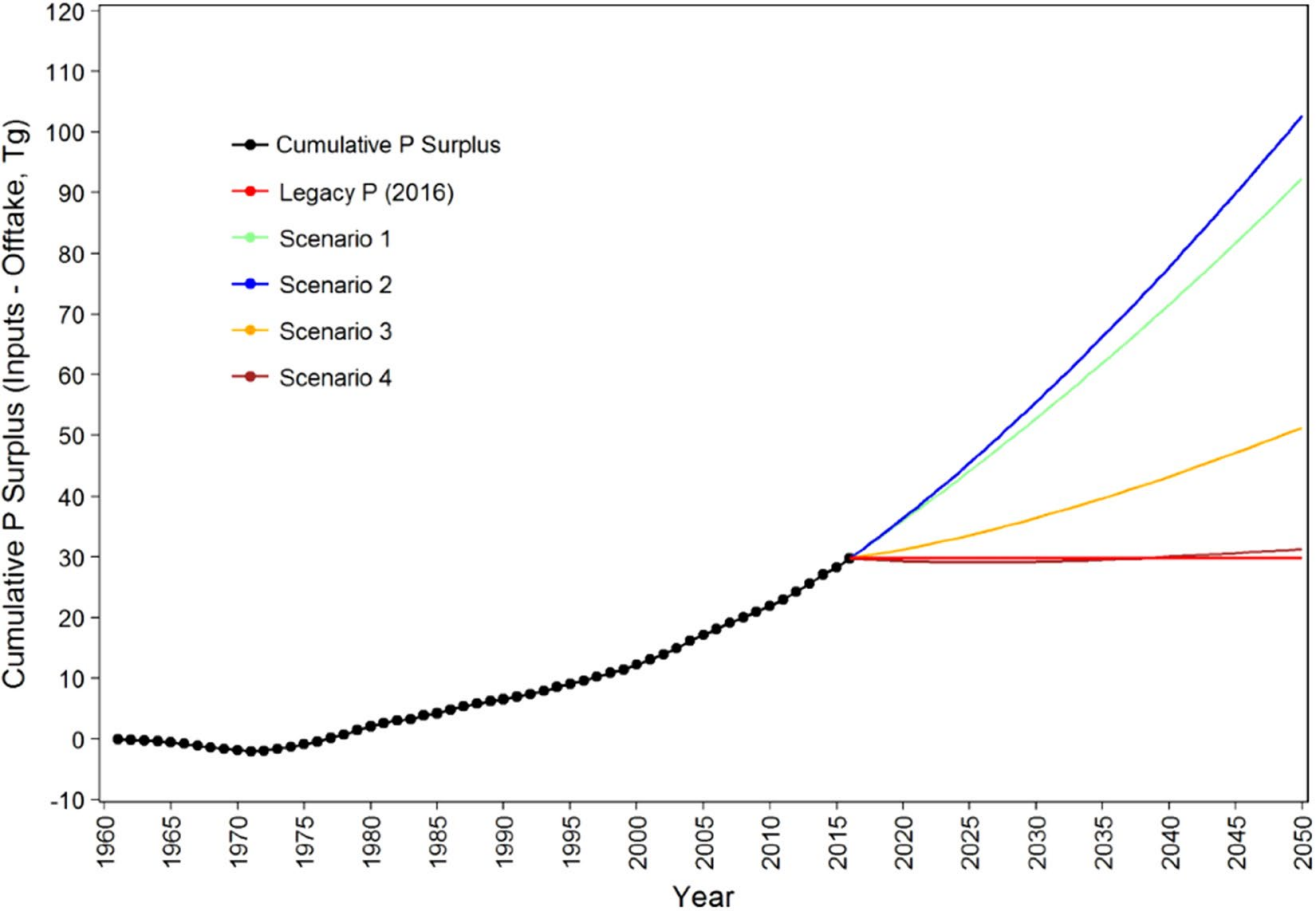
Total legacy P present in Brazilian soils from 1960 to 2050 for each scenario. A P input strategy of applying 50% of plant P offtake (scenario 4) is needed to limit further accumulation of P surpluses in the soil at current cropland expansion rates in Brazil. Scenarios 1–4 are as described in Fig. 1.

Overall, Brazilian agricultural fields have received more fertilizer P than required for crop needs since 1970 (Fig. S3A) and has therefore accumulated significant legacy soil P reserves. We calculated that a cumulative total of over 45.7 Tg of inorganic fertilizer P has been applied in Brazil since 1960, when fertilizers were first regularly used, and that 22.8 Tg of this input remains in the soil. Moreover, an additional 7 Tg of manure P from pig and poultry farms has been recycled to cropland since 1974 based on numbers of animals and their average excreta P content, and assuming all manure is recycled in some form to land (data for confined cattle are available only from 2000^27^). Hence, although a slight underestimate of total manure P loading, the manure input from pigs and poultry represents at least 16% of the total fertilizer P inputs since 1960 (Fig. 1C). Manure inputs therefore make a significant contribution to the surplus P accumulating in Brazilian farming systems and soils but their use lowers the national P efficiency index (defined here as ratio of P inputs in fertilizers and manures to P outputs in crop P offtake), (Fig. 2D). After accounting for cumulative crop P offtake, the total cumulative surplus P accumulating in Brazil’s soils as legacy P since 1960 therefore amounts to at least 29.8 Tg (Fig. 3), with the vast majority of this residing in cropland, since very little P is applied to pasture in Brazil. We estimated the corresponding cumulative amounts of legacy P remaining in the soil by the year 2050 could reach 92 and 103 Tg, respectively for each cropland intensification scenario (Fig. 3). These reserves of P are substantial and in principle could potentially meet Brazil’s crop P demand for many years depending on its bioavailability.

To evaluate the potential crop availability of this legacy soil P, we examined soil P dynamics in six long-term trials (14–38 years) representing typical acidic, clayey, highly P fixing (high Fe levels) soils of the Brazilian Cerrado region, where most of the future agricultural expansion and intensification is anticipated to occur. All site details are given in Table S1. In summary, the sites had soybean, maize and cotton as the main crops during the summer, and cover crops or legumes as the winter crop. At sites 1–5, no tillage (NT) was compared with conventional tillage (CT) systems, but site 6 was cultivated only under a NT system. To compare the accumulation of legacy soil P forms, an adjacent area of native vegetation (NV) close to the experiments, with similar soil type, was also included as a natural reference system (except site 6). At site 6, a high P input system (HP) was compared with a low P input system (LP).

**Figure 4:**
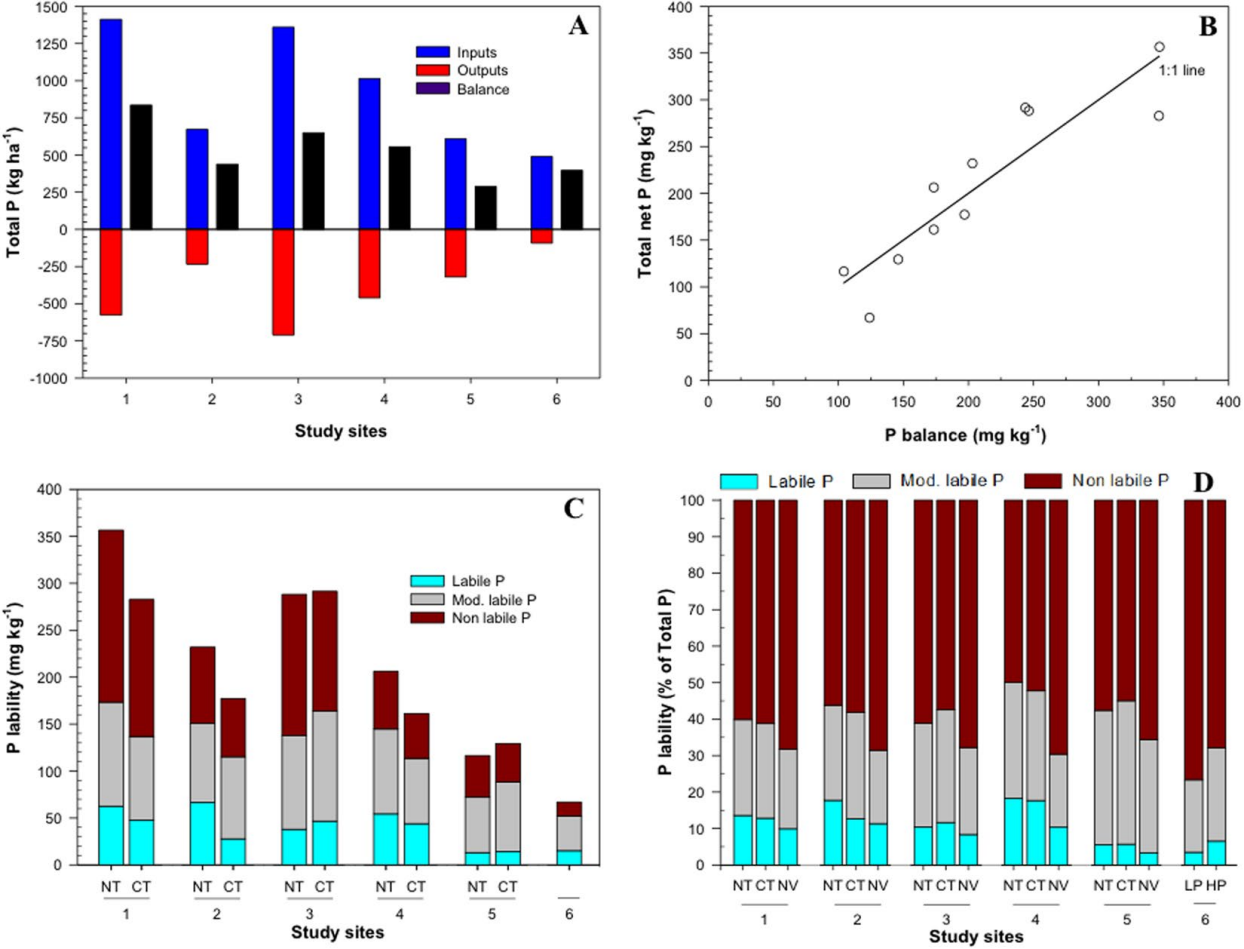
Legacy soil P in six different Brazilian long-term field sites; (**A**) P inputs, outputs and balance since conversion from native Cerrado vegetation; (**B**) Change in soil total P as a function of the total P surplus balance with the 1:1 line drawn; (**C**) Increase in soil P for each tillage/P input treatment as a function of P lability according to the Hedley sequential fractionation method; (**D**) P lability as a percentage of total soil P in native vegetation and cropped areas for each tillage/P input treatment. NT – no tillage; CT – conventional tillage; NV – native vegetation, LP – low P; HP – high P.

A detailed P balance for each site showed that the net P balances accumulating in the soils at each site were fully accounted for by the changes in soil total P between cultivated treatments and the native Cerrado control (Fig. 4A,B and Table S2). Soil total P typically increased up to two-fold as a result of the cumulative surplus P additions under cultivation (Table S3). Of this total P increase, 11–26% was in labile forms (crop available), 32–58% was in moderately labile forms (partly crop available) and 22–52% was in non-labile forms (crop unavailable) according to the Hedley fractionation procedure (Table S3)^40^. More non-labile P tended to accumulate at sites 1 and 3 (Fig. 4C), which had greater clay and Fe oxide contents, and more P tended to accumulate in inorganic P forms than in organic P forms (Table S3). At all sites, and despite continuous fertilizer inputs for up to 38 years, the majority of total P in the soils (i.e. legacy P + native P) still resided in a non-labile form (Fig. 4D). However, the amounts of labile and moderately labile legacy P potentially available to crops still represent a considerable P reserve (up to 7.7 and 15.5 Tg, respectively).

### Transitions to more sustainable phosphorus use

With limited potential to substitute secondary bioresources for annually imported P fertilizer, more radical strategies for improving the efficiency of P use must place a greater reliance on the value of legacy soil P in Brazil^41,42^. Sorption theory suggests soil P immobilization should gradually decrease (i.e. P tax diminishes) as P fixation sites in the soil become blocked leading to an increase in both soil labile P and the P efficiency index^43^. For example, in the long-term sites 1–4 (Table S1), the P efficiency index in maize-soybean rotations gradually increased from 0.47 to 0.80 over the experimental period, and with a notable increase in 2008 when fertilizer inputs were reduced due to excessive pricing of PR on the world market (Fig. S4C). Continued legacy soil P accumulation should therefore eventually increase soil labile P sufficiently to enable a transition to a near-maintenance fertilizer strategy that aligns P inputs more precisely to actual crop offtake^44^. Field trials in Brazil suggested that the combined use of NT, cover crops, correction of acidity and adoption of the 4 R principles of nutrient management (Right rate, Right time, Right place and Right form^45^), could reduce inorganic fertilizer P inputs from their current high level to a near maintenance level to improve overall P use efficiency^46,47^. The current farmer practice of generous overuse of P may therefore not be justified. We estimated that a transition to maintenance P applications (from all annually available P sources) on existing cropland from 2016 onwards (Scenario 3) would reduce the annual surplus in 2050 to 0.83 Tg (i.e. a reduction of 65%), and limit the cumulative P surplus in the soil to 51.3 Tg, or nearly half the corresponding estimates for scenarios 1 and 2 (Fig. 3). This suggests that technology transfer will need to play an important role in the future adoption of more sustainable P input strategies by farmers in Brazil.

Since a significant proportion of the increase in legacy soil P is in labile or moderately-labile forms, we argue here that legacy soil P could further be relied upon to buffer the economic impact of future PR price volatility by applying less P than crop P offtake. It maybe possible to eliminate or reduce further P applications if there is sufficient available legacy P to maintain soil P supply and prevent any decline in crop yield. Recent evidence for a clay soil under NT management in Southern Brazil suggested that moderately labile P was at least partially utilized by crops (Table S4). Other field evidence suggested that the recovery of legacy P could be as high as 80% depending on cropping system and the amount of available legacy soil P present^46^. However, it is difficult to predict the bioavailability of legacy soil P, and, at this point, total reliance on legacy soil P to supply sufficient crop P is risky. Although unfertilised areas with high levels of legacy P have generally shown yield losses of soybean and maize, as compared with fertilized areas, an intermediary P input strategy with plant demand being in part supplied by the soil P reserve could be achieved without yield loss^46^. In support of this strategy, a range of recent studies in China have shown how crop yield can be maintained, or even increased, while fertilizer application is reduced below offtake requirements by targeting the P supply to the crop root zone to increase P uptake efficiency^48^. We estimated that a transition to 50% below-maintenance P applications on existing cropland from 2016 onwards (Scenario 4) would be needed to limit the cumulative P surplus in 2050 to close to its 2016 level of ca. 30 Tg (Fig. 3).

## Discussion

The investment cost of increasing soil P fertility to optimize crop yields in Brazil is high because of the P fixation tax demanded by Cerrado soils following conversion to cropland^5,14^. Fertilizer P inputs are still double crop P offtake nationally (Fig. 2D), and Roy *et al*.^14,49^ argue that this high rate of P input will need to continue for many years, especially on clayey soils. This P input strategy may not be sustainable in the long-term if Brazilian cropland continues to expand at its current rate of 2.6% yr^−1^, and continues to rely on imports of primary fertilizers derived from finite global P reserves. Brazil’s own reserves of exploitable PR are of igneous origin and of relatively poor quality (i.e. low P content and low solubility in citric acid) compared to the higher quality sedimentary-derived PR currently imported from Morocco, Tunisia, Algeria, Egypt, USA and Peru^50^. Total reserves of PR currently available for exploitation in Brazil are estimated at 320 Mt^51^, which at current extraction rates (ca. 6.7 Mt yr^−1^) will be exhausted in about 50 years. However, investment in geological research over the last decade has identified new potential PR reserves in Brazil that are not yet accounted for, although their quality and the costs to process them remain uncertain. Investments in new PR processing technologies will be needed to capitalise on these PR resources of marginal quality, and the price of PR on the world market (driven by PR supplies from Morocco) will determine decisions on new mine investments^19^. Brazilian agriculture is therefore likely to remain heavily dependent on inorganic P imports to satisfy its growing fertilizer P demand and therefore vulnerable to future P scarcity. Continued overuse of P fertilizer relative to crop demand will also lead to further soil P accumulation and potential long-term eutrophication problems^52,53^.

Secondary bioresources in Brazil could make an important contribution to annual P fertilizer demand, provided these materials are available to apply in both existing and expanding frontier areas, and have adequate P bioavailability. Brazilian farmers will require evidence that these materials are satisfactory alternatives to highly soluble mineral fertilizers that they have come to depend on. For example, research suggests that while filter cake is an effective nutrient source^38^, the P availability of Fe-rich wastewater biosolids may be more limited^54^. Alternative biotechnologies to treat human and industrial wastewater will be necessary to continually provide a renewable resource which is of better value to soil quality and nutrient provision in Brazilian soils. Our analysis also highlights a major underinvestment in recovering P from the food chain in Brazil. If 80% of Brazil’s 210 million people were connected to a central sewerage collection system (over 80% of Brazil’s population live in cities), and 80% of this collected waste was treated, and 80% of the biosolids produced was recycled to land, we estimate a potential secondary resource of 10.3 Gg of P annually. This is over ten times more than is currently recycled. Similarly, food waste in Brazil will be considerable, but this resource is currently not being conserved and recycled. Assuming food waste per person in Brazil is the same as in Europe (173 kg)^55^, and that food waste contains approximately 1.5 g P kg^−1^ of waste^56^, this bioresource could provide 45 Gg of P annually. Developing an effective circular economy for P in Brazil will therefore require further investment in P recovery infrastructure, and the creation of suitable markets for these secondary P resources, including those linked to the production of bioenergy, although this will remove the value of the organic matter present in the original material^57–59^. For example, (partial) recovery of P from different bioresources would enable their more widespread distribution. With additional investment in P recovery technologies for wastewater and food waste (as discussed above), and using all potentially available secondary bioresources as detailed in Table 1, we estimate that annually produced bioresources could provide no more than ca. 0.68 Tg of P by the year 2050, or 15–20% of projected fertilizer demand. Although a potentially significant and nutritionally useful secondary P resource, the fertilizer substitution value of annually produced bioresources in Brazil is therefore limited in capacity.

The largest potential source of secondary P that might be used as a substitute for fertilizer P imports is clearly the legacy soil P that has already accumulated in Brazilian soils (28 Tg). This secondary P resource can be accessed *in situ* and incurs no external costs of recovery. The utilization of legacy soil P in existing cropland areas is attractive because it not only provides immediate financial savings on inputs of inorganic P fertilizers, but also reduces the longer-term risk of water eutrophication arising from P in land runoff^42^: for example, on more steeply sloping land converted from pasture where erosion risk might be expected to be greater. Our estimate of legacy soil P is greater than the 20 Gg estimated by Roy *et al*.^14^ who accounted for mineral fertilizer P inputs only, and used FAOSTAT land areas^60^ rather than Brazilian census data. At a current P fertilizer price of $1.4 kg^−1^ of P, this legacy P is valued at over $40 billion if it could all be utilized by crops. Regional variation in soil legacy P will be large, especially in areas where livestock manures cannot be distributed evenly due to transport costs, and where they are applied to crops to meet their nitrogen (N) requirements due to a low manure N:P ratio. The strong relationship between surplus P accumulation and soil total P across the field sites reported here (Fig. 4B) suggests that simple measurement of soil total P in cropped and naturally vegetated areas will provide a good guide to the total legacy P reserves present.

The major barrier to the use of legacy P in tropical soils for profitable crop production is whether it can be mobilized sufficiently to provide all or part of crop P demand, and over what time period this soil P store can be utilized. While labile P fractions can be assumed to be fully crop available, the fertilizer replacement value of moderately labile and non-labile P forms of legacy soil P for Brazilian farming systems requires further clarification. However, just considering the significant amounts of labile P remaining in the soil, there is clear potential value in utilizing legacy soil P as a buffer against future P scarcity, or fluctuating P prices, by allowing short-term cessation of P inputs without risk of yield penalty. In the longer-term, sustainable P use and increased resilience of food production systems in Brazil, as elsewhere, will require crop production systems to be redesigned to lower crop P demand, operate under lower soil P fertility and maximise soil P acquisition and P use efficiency through advanced crop, microbial and fertilizer engineering – termed agro-engineering by Rowe *et al*.^42^. This redesign could be achieved through the development of more integrated farming systems linked to land use capability^61^, more P-efficient crop cultivars with lower seed total P^62^, crop rotation design to exploit the plants innate ability to scavenge for soil P^63^, the use of targeted bio-inoculants^64^, and the development of novel fertilizers that by-pass the soil^65^ (See further supplementary discussion on Agro-engineering).

In summary, there is large scope to expand cropland output in Brazil without impeding the nation’s extensive grazed beef production systems or contravening deforestation rules. Brazil’s high P fertilizer demand leads to large inefficiency because it does not account for legacy soil P reserves. Crop production to meet demand will continue to represent a drain on global P resources unless more sustainable P input strategies are adopted. These strategies should include (a) national and regional investment in P mining and P recovery technologies to provide cost-effective domestic PR sources, or secondary P resources that can substitute for imported P, and (b) transition to maintenance, or below maintenance, P input strategies where legacy soil P stocks provide all, or a portion, of crop P requirements and reduce reliance on diminishing PR reserves. With a bank of soil P fertility for long-term P security, farming systems could be redesigned to perform profitably and efficiently under lower levels of soil P fertility.

## Methods

### Intensification Scenarios

Two scenarios for agricultural intensification in Brazil to 2050 were constructed based on past trends and national census data of Brazil’s total cropland area^27^, crop production^66^, animal numbers^27^, and P fertilizer consumption^67^.

#### Scenario 1

Brazil’s agricultural intensification will occur only on the existing cropland area and crop yields will increase by an average 1.5% per year based on the combined average yield trends of the major crops maize, soybean and sugarcane over the last twenty years.

#### Scenario 2

Brazil’s agricultural intensification will occur on the existing cropland area *and* new croplands (i.e. excluding commercial forests and pasturelands) will expand into both native Cerrado areas and degraded pasture areas at a combined rate equivalent to the increase in the annual cropland area over the last 20 years (2.6% yr^−1^).

#### Cropland and crop production

Total cropland includes all annual temporary crops and permanent crops, but since the permanent cropland area has remained very stable (Fig. S1A), cropland expansion to 2050 was based on the rate of change in annual cropland area over the last twenty years (1996–2015). Trends in crop yields were based on the major crops maize, soybean and sugarcane, which represent 82% of all annual crops in Brazil (Fig. S1B), and where most of the future agricultural expansion will occur. The predicted expansion of these crops to 2050 was based on average yield trends (1996–2015 Fig. S2). This was a period of improved economic stability in Brazil and provides a robust base for future forecasts^30^. Yield trends were estimated using the autoregressive (AR) integrating (I) moving-average (MA) model (commonly named ARIMA), accounting for autocorrelation in the time-series, selected based on Akaike information criteria (AIC)^68^. The time-series analyses were performed in R 3.4^69^, using the forecast package^70^. The annual yield increment trends for each of the three crops were normalised for yield level and pooled by the parametric bootstrap method^71^ to provide a statistically-based combined average yield trend, which was then applied to all cropland.

#### Animal numbers and manure P production

Trends in numbers of total cattle (dairy + beef), pigs and poultry (data available from 1974^27^) were based on incremental rates over the last decade (2006–2015): cattle, 0.38%, pigs, 1.70%, layer hens, 1.76% and broiler chickens, 4.17% (Table S1). Amounts of P excreted by grazing cattle were assumed to be totally recycled to pasture areas and were not included further. Data on confined cattle were not included in the trend analysis for manure P production (Fig. 2B) because future expansion of livestock sector is forecast to be largely in pig and poultry numbers^30^. However, manure P from confined cattle is included in the bioresource estimates for 2016 and 2050 (see below). Average amounts of P excreted by pigs and poultry annually were based on the typical volumes of excreta produced by each type of livestock and average values of P in excreta modified to take account of larger liveweights in Brazil. For pigs, we assumed a daily excretion of 7 L day^−1^ of slurry with a density of 1.014 kg dm^−3^ and P content of 0.91 g P kg^−1 ^^72^. For poultry hens, we assumed an annual excretal P production of 0.24 kg P animal^−1^ yr^−1 ^^73^. For litter from broiler chickens, we assumed a standard chicken house with 20,000 birds and six production cycles of 60 days would generate 220 Mg of litter in each cycle with a P content of 9.3 g P kg^−1^. All P excreted by pigs and poultry is assumed to be recycled to cropland areas.

#### Fertilizer P consumption

Trends in total fertilizer P use (data for deliveries on farm available from 1960^67^) to 2050 for scenario 1 assumed that the P fertilizer requirement will increase proportionally to crop yield (i.e. 1.5% yr^−1^). This maybe an overestimation in practice if farmers are economically constrained and limit P applications within the next 35 years. For scenario 2, fertilizer P inputs to the existing cropland area were assumed to the same as for scenario 1, but for new areas we assumed an initial single input of 35 kg P ha^−1^ to overcome soil P fixation^25^, followed by a constant input of 25.2 kg ha^−1^ which is the average annual fertilizer P use on crop land over the last 10 years.

#### P surpluses and efficiency index

P surpluses for each scenario were calculated as the difference between the total P inputs (fertilizers + manure) and total P outputs (crop offtake) and did not include P losses in surface runoff which were anticipated to be minimal in a P-fixing soil environment^49^ and flat cerrado landscapes. Total P inputs were calculated as above. Annual average crop P offtake was calculated for both annual and permanent crops based on data from Roy *et al*.^14^. Predictions of crop P offtake for the years 2016–2050 assumed that P offtake would increase at the same rate as yield (1.5% per year), and that crop yields in new areas (Cerrado and degraded pasture) would be the same as the national average when P fertilizer was applied. A P efficiency index was calculated as the ratio of P inputs (fertilizer + manure) and crop P offtake.

### Secondary P Resources in Brazil

The amounts of P contained in potential secondary P resources that could be used as a substitute for mineral fertilizers derived from PR were calculated for the year 2016 and projections made to the year 2050.

#### Livestock manures

Estimates of P in pig and poultry manure were made as detailed above. It should be noted that national averages conceal large variability in the P content of livestock diets on individual farms and this can influence local excretal P concentrations. Using current values of manure P content may also underestimate manure P loadings in earlier years because modern diets that include phytase lead to lower excretal P contents. Numbers of confined cattle in 2015 and 2050 assumed that 15% of total cattle are slaughtered annually^27^ and 10% of slaughtered cattle are confined feedlot cattle (according to ANUALPEC, cited by Costa Junior *et al*.^74^). Export of P in cattle excreta was taken as 6 kg P animal^−1^ yr^−1^ assuming a medium P diet^75^.

#### Sugarcane

P contained in the filter cake produced as a by-product from the processing of sugar cane assumed 5 kg of cake is produced per tonne of sugar cane harvested with a P content of 10 g kg^−1 ^^38,76^. We assumed there would be no transport restriction of filter cake to a sufficient area of agricultural land for recycling up to 2050 and a long-term P bioavailability of 100%, as is usually assumed in fertilizer recommendation systems when assessing the value of bioresources to long-term soil P fertility^77^.

#### Wastewater biosolids

P contained in biosolids assumed that 48% of the population were connected to a sewerage collection system, 66% of collected sewage was actually treated, 12 kg of biosolids (100% dry matter) were produced per capita of the treated population, and 15% of the biosolids produced after treatment were recycled to land with a P content of 8.5 g P kg^−1^ biosolid DM^37^. Population estimates for 2050 were taken from the United Nations database^78^.

#### Legacy soil P

Legacy P remaining in the soil since 1960 was taken as the cumulative surplus calculated from the balance between P inputs (fertilizers and manures) minus crop P offtake as calculated above.

Six long term trials in Brazil’s main cropland production areas were selected to investigate the amounts and forms of legacy soil P that have accumulated since conversion of the native Cerrado vegetation. A summary of the background site details is given in Table S1. At sites 1–5, soil P was measured under no-tillage (NT) and conventional tillage (CT) treatments with different cover crops and compared with soil P under natural cerrado vegetation (NV) as a reference area^15^. At site 6 under a NT cultivation system, soil P was measured for treatments comparing low P (LP) fertilizer (kg 7 P ha^−1^) and high P (HP) fertilizer (kg 35 P ha^−1^) inputs. The experimental treatments were arranged in a randomised block with three replicates. These sites are representative of a large area of cultivated cropland in the main Cerrado region of Brazil, with acid, clayey, high P-fixing soils and typical cropping systems that include soybean, maize and cotton. They provide a suitably large range in the legacy period over which a P input-output balance can be calculated (14–38 years) (Table S1).

For each site, a detailed P balance was estimated for each treatment considering the total fertiliser P inputs and the total P offtake in the harvested crop since the area was converted from the native forest vegetation to cropland (Table S2). Annual P inputs (fertilizer P) and P outputs (yield × grain P content) were based on measured values for the period of the replicated trial, and estimated for the period between deforestation and when the trial began (when measured data were not available) by considering the average values from the first three trial years (sites 1, 3 and 4). Soil samples to a depth of 20 cm were collected from all treatments and natural reference areas at each site by auger in 2011 (site 5), 2012 (sites 1 and 2), 2013 (3 and 4) and 2015 (site 6) for P soil P analysis. Previous work showed that P did not migrate below this depth^15^. Changes in soil P forms were determined by the Hedley sequential P fractionation method^79^, with modifications by Condron *et al*.^40^. This method uses chemical extractants to remove progressively from the same sample the most available to the most stable forms of inorganic (Pi) and organic P (Po). Sequentially-extracted P was grouped into three fractions of P lability: a) labile P, corresponding to the inorganic P extracted by anion exchange resin and the inorganic and organic P extracted by 0.5 mol L^−1^ NaHCO_3_; b) moderately-labile P, corresponding to the inorganic and organic P extracted by 0.1 mol L^−1^ NaOH, and inorganic P extracted by 1.0 mol L^−1^ HCl; and c) non-labile P, corresponding to the inorganic and organic P extracted by 0.5 mol L^−1^ NaOH, and the P in the remaining residue^15^. The soluble reactive P concentration in each extract was measured according to colourimetry using the Murphy and Riley^80^ procedure for acid extracts, and Dick and Tabatabai^81^ procedure for alkaline extracts. Organic P in each extract was determined by the difference between total P and inorganic P. A summary of the amounts of P extracted in each sequential P fraction is given in Table S3.

### Sustainable P management scenarios

Two scenarios of more sustainable P management in Brazil up to the year 2050 were devised (Scenarios 3 and 4) to examine their potential impact on fertilizer P demand and surplus soil P accumulation compared to the previously discussed scenario 2, which is the most likely to occur and includes both intensification of existing cropland and cropland expansion into native Cerrado and degraded grassland.

#### Scenario 3

Total annual P inputs (all sources) into existing cropland in Brazil will not exceed annual crop P offtake. This scenario assumes that adequate levels of soil labile P for optimum yields in Brazil have already been attained. Surpluses of P will still occur in this scenario because of P inputs to overcome P fixation in new frontier areas converted from native Cerrado, or degraded pastureland.

#### Scenario 4

Total annual P inputs (all sources) into existing cropland in Brazil will not exceed 50% of annual crop offtake. This scenario was developed to produce no further increase in the cumulative legacy P in the soil in 2050. It represents a more extreme P input strategy that relies on the bioavailability of legacy soil P reserves to sustain yields and still needs to be proven feasible.

## Electronic supplementary material

Supplementary Information
