# Supplementary material for: Transitions to sustainable management of phosphorus in Brazilian agriculture
Source: Sci Rep. 2018 Feb 7;8:2537. doi: 10.1038/s41598-018-20887-z (PMC5803245; doi:10.1038/s41598-018-20887-z)
Supplement: Supplementary file 1 — Supplementary Information [file 41598_2018_20887_MOESM1_ESM.pdf]

Transitions to sustainable management of phosphorus in Brazilian agriculture

Paul J. A. Withers[1*], Marcos Rodrigues[1,2], Amin Soltangheisi[2], Teotonio S. de Carvalho[3], Luiz R. G. Guilherme[3], Vinicius de M. Benites[4], Luciano C. Gatiboni[5], Djalma M. G. de Sousa[6], Rafael de S. Nunes[6], Ciro A. Rosolem[7], Fernando D. Andreote[2], Adilson de Oliveira Jr.[8], Edson L. M. Coutinho[9], Paulo S. Pavinato[2]

[1]School of Environment, Natural Resources and Geography, Bangor University, Thoday Building, Bangor LL57 2UW, UK.

[2]College of Agriculture 'Luiz de Queiroz', University of São Paulo - ESALQ-USP. Av. Pádua Dias, 11. CEP: 13418-900. Piracicaba, SP, Brazil.

[3]Federal University of Lavras - UFLA. Campus Universitário. PO Box 3037. CEP 37200-000 - Lavras, MG - Brazil.

[4]Brazilian Agricultural Research Corporation, Embrapa Soils. Rua Jardim Botânico, 1024. CEP 22460-000. Rio de Janeiro, RJ, Brazil.

[5]Santa Catarina State University - UDESC. Av. Luís de Camões, 2090. CEP 88520-000 Lages (SC), Brazil.

[6]Brazilian Agricultural Research Corporation, Embrapa Cerrados. BR 020, Km 18 Planaltina. PO Box 08223. CEP 73310-970 - Brasília, DF - Brazil

[7]São Paulo State University, FCA/UNESP. Rua José Barbosa de Barros, 1780. CEP 186010-307. Botucatu, SP – Brazil

[8]Brazilian Agricultural Research Corporation, Embrapa Soybean. PO Box 231. CEP 86001-970. Londrina -PR– Brazil.

[9]São Paulo State University, FCAV/UNESP. Via de acesso prof. Paulo Donato Castellane, s/n. km 5. CEP 14884-900. Jaboticabal, SP – Brazil

*Correspondence to p.withers@bangor.ac.uk

Supplementary Material

1. Brazil's Green Revolution

Brazil has a total land area of approximately 243 M ha devoted to food production, of which ca. 166 Mha (68%) are currently in managed pasture, and ca. 77 M ha are managed cropland[1,2]. Annually cultivated crops have increased rapidly since the mid 1990's when Brazil became economically more stable, while permanent crops (e.g. commercial forest and orchards) have remained constant (Fig. S1A). The three main cultivated crops are soybean (~31 Mha), maize (~15 Mha) and sugarcane (~9 Mha), which together account for 82% of Brazil's annual crops (Figure S1B). The areas of these three main crops have steadily expanded over the last 40 years, but with soybean showing a much steeper rise since 2000 (7.5% yr^{-1}) in response to a greater demand for export to Asia.

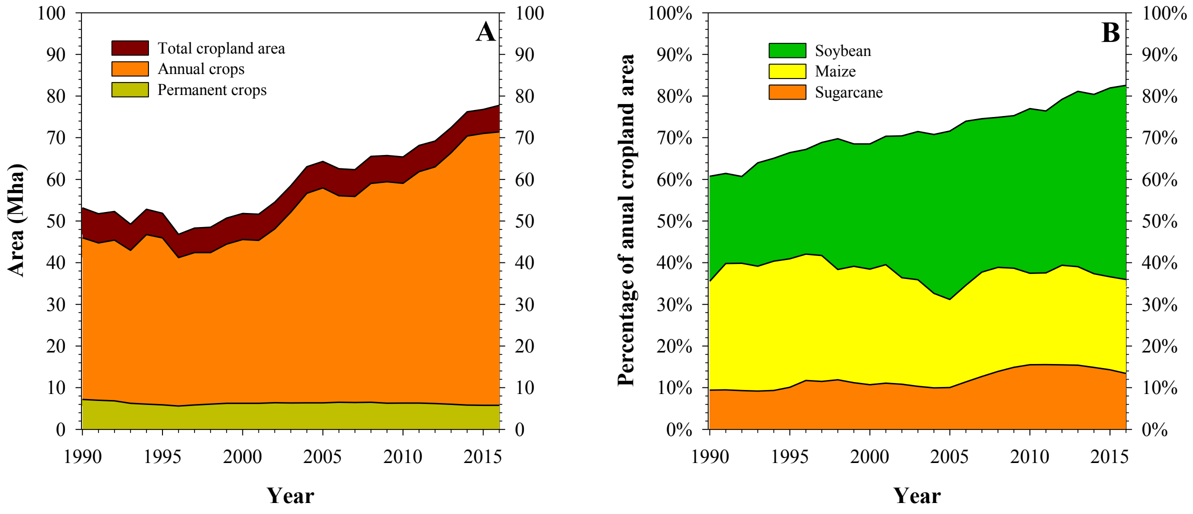

Figure S1. Trends in Brazil's cropland area from 1990 to 2016: **A)** Total, annual and permanent cropland area; **B)** Soybean, maize and sugarcane area as a percentage of the total annual cropland area.

Average crop yields and total production have also increased dramatically over this period: from 1.6 to 5.7 t ha^{-1} for maize, 1.3 to 3.1 t ha^{-1} for soybean and 37 to 73 t ha^{-1} for sugarcane (Fig. S2). Soil quality improvements resulting from the gradual conversion to no tillage cultivation systems, which now account for 54% of the total cultivated area in Brazil, have also contributed to increased crop production[3,4]. In contrast to the cultivated cropland area, the area of pasture for cattle production in Brazil has remained relatively stable; for example from 154 Mha in 1974 to 166 Mha in 2006[1]. Stocking rates and soil fertility in Brazil's extensive pasturelands still remain relatively low and well below their productivity potential[5].

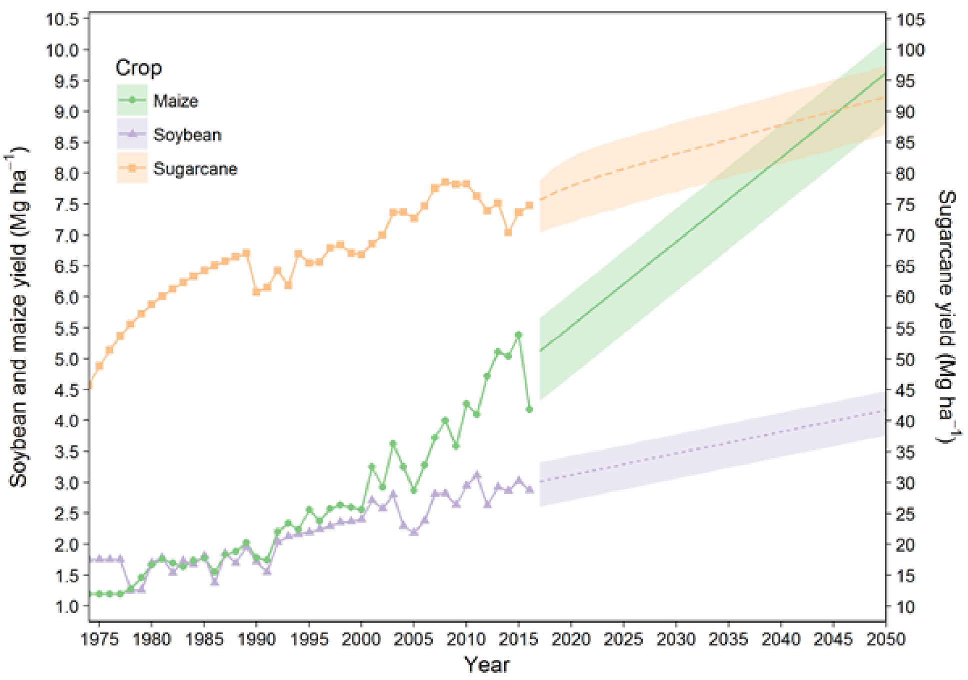

Figure S3. Current and future (2017-2050) trends in Brazil's soybean, maize and sugarcane yields. Coloured bands represent the uncertainty surrounding future predictions.

2. Phosphorus fertilizer use

Phosphorus fertilizers were regularly applied in Brazilian agriculture after 1960, but their use increased more rapidly after 1990 (Fig 3A). Initially, P inputs did not match crop offtake, but since the mid 1970's, fertilizer P rates have exceeded P offtake in harvested product by a factor of 2 (Fig. 3A). As the cropland area has also expanded rapidly into the native Cerrado and degraded pastureland in recent years, average rates of fertilizer use over all cropland have stabilized and are currently 25-28 kg P ha^{-1} yr^{-1} (Fig. S3B).

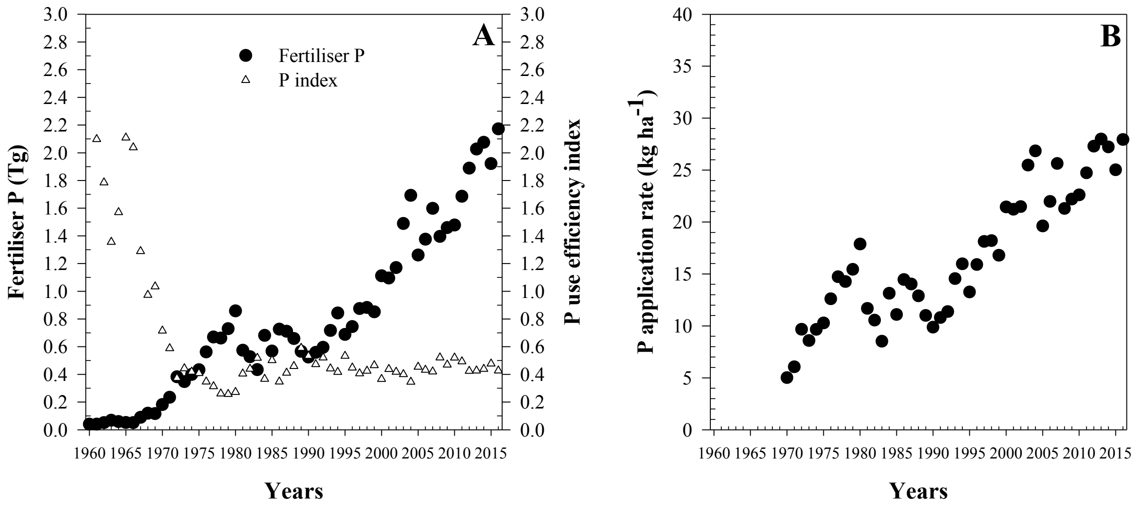

Figure S3. Total consumption of P fertilizers (all land uses) in Brazil and their P efficiency index **(A),** and the average annual P application rate to all cropland **(B)** from 1960-2016. The P efficiency index is the ratio of total cropland P offtake to total P fertilizer use.

3. Legacy soil phosphorus

Data tables for the six long-term field experiments assessing total amounts of legacy P in cerrado soils representative of the main cropping areas in Brazil, and an assessment of legacy P bioavailability. Discussion of a P efficiency index (Figure S4) is also shown.

Table S1. Site details, soil characteristics (0-20 cm) and cropping system for the six long-term experiments.

Parameter	Site 1	Site 2	Site 3	Site 4	Site 5	Site 6
Location (City-State)	Costa Rica - Mato Grosso do Sul	Sapezal – Mato Grosso	Cristalina - Goiás	Tasso Fragoso – Maranhão	Planaltina - Distrito Federal	Botucatu - São Paulo
Biome	Cerrado	Cerrado	Cerrado	Cerrado	Cerrado	Atlantic Forest/ Cerrado
Rainfall[1] (mm)	1937	2156	1606	1476	1570	1450
Altitude (m)	790	640	981	610	1014	840
Soil Order[2]	Oxisol	Oxisol	Oxisol	Oxisol	Oxisol	Oxisol
Clay (%)	65.6	45.2	65.3	25.5	64.0	22.0
Silt (%)	24.3	36.3	22.3	4.6	9.0	11.0
Sand (%)	10.1	18.5	12.4	69.9	27.0	77.0
pH[3]	5.4	4.8	5.1	4.6	4.7	4.9
Organic C (%)	1.91	1.84	1.50	1.05	1.62	1.42
Fe ($g\ kg^{-1}$)[4]	48.8	48.1	27.4	19.8	nd	17.9
Al ($g\ kg^{-1}$)[4]	10.7	13.1	12.5	5.8	nd	nd
Cultivation system	No-till and conventional	No-till and conventional	No-till and conventional	No-till and conventional	No-till and conventional	No-till
Cropping rotation	Soybean, cotton, cover crops	Soybean, cotton, cover crops	Soybean, maize, cover crops	Soybean, maize, cotton, cover crops	Soybean, maize, cover crops	Soybean, brachiaria (cover crop)
Treatments used	Tillage management	Tillage management	Tillage management	Tillage management	Tillage management, P sources/ methods	P rates and sources
Date started	1974 (deforestation) 1994 (trial began)	1997 (deforestation) 2001 (trial began)	1977 (deforestation) 1992 (trial began)	1990 (deforestation) 2001 (trial began)	1976 (deforestation) 1994 (trial began)	2001 (trial began)
Legacy period (years)	38	15	36	23	17	14

[1]Long-term (last 30-year) annual average; [2]USDA soil classification system; [3]$CaCl_2$; [4]Dithionite-citrate-bicarbonate (DCB); nd: not determined.

Table S2. Estimated P balance (inputs, outputs and surplus) in six long-term sites representative of Brazil's main crop production areas using either no-tillage (NT) or conventional tillage (CT) cultivation systems (sites 1-5), and low P (LP) or high P (HP) fertilizer inputs (site 6).

Phosphorus Balance	Sites											
	1		2		3		4		5		6	
	NT	CT	NT	CT	NT	CT	NT	CT	NT	CT	LP	HP
	---------- kg P ha^{-1} ----------											
Total P input	1412	1412	672	672	1359	1359	1013	1013	608	608	96	489
Total P output	576	577	227	240	706	713	460	460	368	272	49	92
Net P surplus	836	835	445	432	653	646	553	553	240	336	47	397
Annual P surplus	22	22	30	29	18	18	24	24	14	20	3	28

Table S3 – Amounts of inorganic (Pi) and organic P (Po) in each soil P fraction (0-20 cm) at each site according to the Hedley sequential fractionation procedure. Treatments were no-tillage (NT) cultivation, conventional tillage (CT) cultivation or natural cerrado vegetation (NV)(sites 1-5), and low P (LP) or high P (HP) fertilizer inputs (site 6).

Site	Treatment	Labile-P		Moderately-labile P		Non-labile P		Total P	
		Pi	Po	Pi	Po	Pi	Po	Pi	Po
		---------- mg kg^{-1} ----------							
1	NT	58.3	42.2	114.8	79.9	406.1	39.2	579.2	161.3
	CT	52.1	33.6	114.8	58.1	359.9	48.1	526.8	139.8
	NV	18.6	19.5	55.2	28.5	249.4	12.7	323.2	60.7
2	NT	70.8	40.9	85.9	78.4	294.6	59.5	451.4	178.8
	CT	42.4	30.5	96.0	71.8	281.9	52.8	420.3	155.1
	NV	24.1	21.0	45.1	35.1	250.4	22.4	319.6	78.5
3	NT	52.9	17.3	79.6	112.4	337.0	74.8	469.6	204.4
	CT	50.7	28.1	77.3	132.0	323.8	65.5	451.8	225.6
	NV	18.9	13.3	32.5	59.5	234.7	26.9	286.1	99.8
4	NT	62.5	13.7	104.6	27.9	186.7	21.6	353.8	63.2
	CT	56.6	9.1	90.5	21.5	170.6	23.8	317.7	54.3
	NV	15.7	6.1	37.0	5.3	139.1	7.7	191.7	19.1
5	NT	12.6	9.7	92.5	54.6	206.9	23.3	312.0	87.6
	CT	13.8	9.6	84.5	77.8	205.4	21.3	303.7	108.7
	NV	1.8	7.5	34.6	53.4	163.7	22.1	200.1	83.0
6	LP	10.2	2.0	26.6	43.0	247.8	20.4	284.6	65.3
	HP	20.5	6.9	40.6	65.9	254.0	29.1	315.0	101.8

At sites 1-4, the change in the annual average P efficiency index (P output/P input) over the experimental period was calculated. When years in cotton cultivation are excluded (sites 1, 2 and 4), the efficiency index gradually increased (Fig S4C) due to the increase in soil labile P, and greater contribution of soil labile P to crop P uptake. The efficiency of P use by the cotton crop is very poor compared to soybean and maize (Fig. S4D).

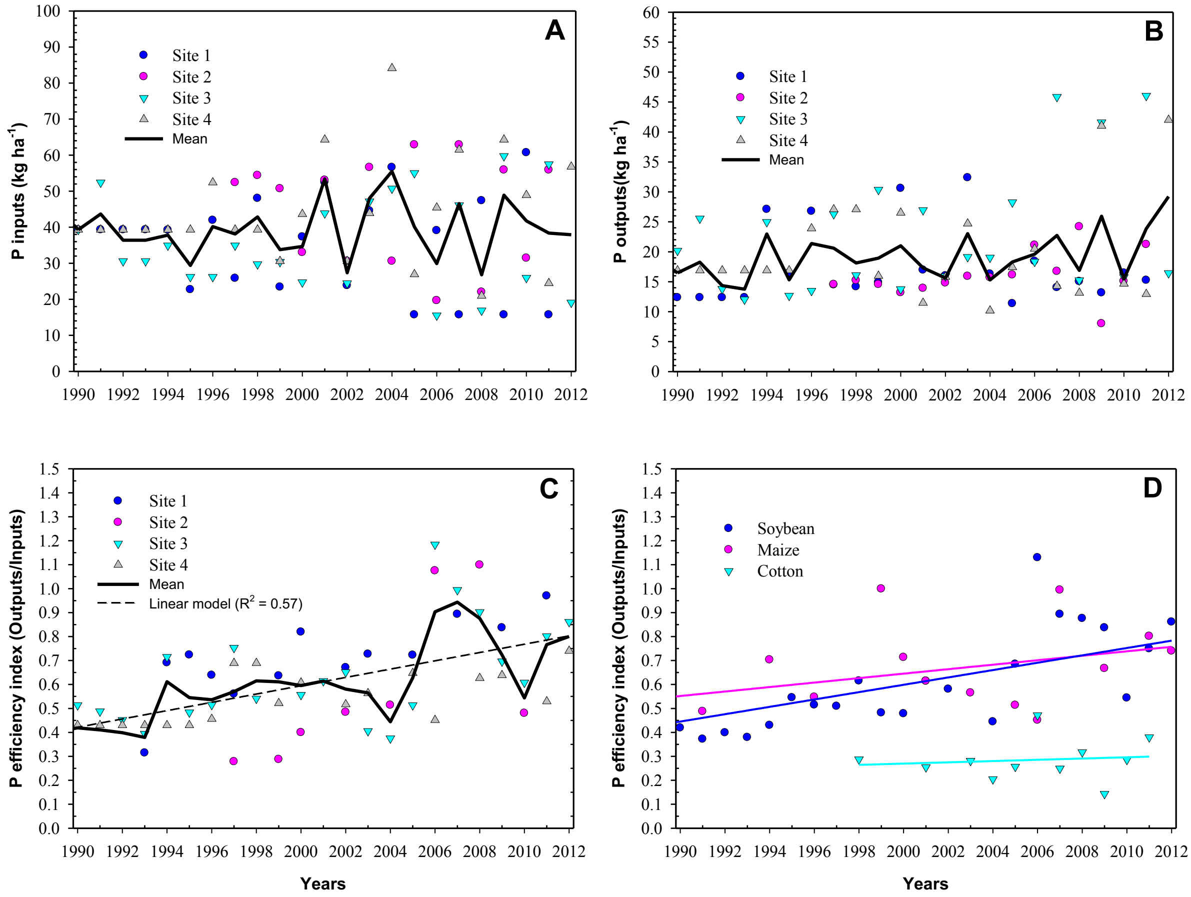

Figure S4. Phosphorus inputs (A), outputs (B) and P efficiency index for soybean and maize crops at long-term sites 1-4 from 1990 to 2012 (C), and the corresponding P efficiency index for each individual crop, including cotton, over the same period (D).

4. Bioavailability of moderately-labile phosphorus

Using a clayey soil under a NT cultivation system that previously received P fertilizer at rates twice the plant needs over 5 five years, Gatiboni et al.[6] found that legacy soil P reserves provided sufficient P to give comparative crop yields over 6 years relative to crops which continued to receive P fertilizer (Table S4). Labile P extracted by anion exchange resin dropped from 30 mg kg^{-1} at the start of the 6 year crop rotation to 21.2 mg kg^{-1} at the end (20 mg kg^{-1} is the critical level of resin P for this soil). Over the same 6-year period, the crops exported 18.7 mg kg^{-1} of P. Soil P fractionation analysis showed that moderately labile P forms also reduced by 24.7 mg kg^{-1} P[7], and additional enzyme assays showed an increase in acid phosphatase activity[8], which may have accelerated the utilization of moderately labile P. These data suggest that P legacy stored in less labile forms can be mobilised when P is withheld.

Table S4. Phosphorus balance after six successive crops in a greenhouse experiment without P applied, using a clayey soil collected under no tillage cultivation system, and previously fertilized for six years with 52 kg P ha^{-1} yr^{-1}.

Parameter	Initial soil P	After six crops[1]	Relative change[2]
Relative yield (%)[3,4]	-	99.3	- 0.7
Cumulative P removal (mg kg^{-1})[4]	-	18.7	- 18.7
Labile P (mg kg^{-1})[5]	30.0	21.2	- 8.8
Moderately labile P (mg kg^{-1})[6]	393.4	370.9	- 24.7
Acid phosphatases activity (mg kg^{-1} hr^{-1})[7]	419.2	618.1	+ 198.9

[1] Six successive crops grown in a greenhouse (around 30 days each), being millet (crop 1), black oat (crops 2, 3, 4) corn (crop 5), soybeans (crop 6).
[2] Difference between values before and after six crops.
[3] Yield as a percentage of plants which received P. Data from: Gatiboni[7].
[4] Amount of soil P removed in total crop offtake over the 6-year crop rotation[8].
[5] Labile P extracted by anion exchange resin[6], where 20 mg kg^{-1} is considered the critical level for this soil.
[6] Sum of moderately-labile P fractions (extracted with 0.5 M $NaHCO_3$, 0.1M NaOH and 0.5 M NaOH)[6].
[7] Data from Gatiboni et al[8]

4. Redesigning production systems through agro-engineering

Crop system engineering: One crop engineering strategy is to grow more P efficient cultivars that store less phytate and total P in their tissues and grain, and therefore remove less P at harvest and require less P in fertilizer[9,10]. The advantages of low P grain also extend into the livestock sector with reduced P excretion rates, and into the human health sector with improved utilization of trace elements (Zn, Fe,) within the body that would otherwise be immobilised by phytate. As nationally ca. 65% of P fertilizer in Brazil is currently applied to soybean, maize and sugarcane[11], a reduction of 25% in the average seed P content (5 kg P/t on soybean and 3 kg P/t on maize) and a decrease of 25% in the average shoot P concentration of sugarcane (5 g/kg DM[12]) would reduce current P fertilizer requirements by 0.76 Tg annually (or 35% of current P fertilizer inputs).

An additional crop breeding strategy to enable a transition to lower P fertility soils is to improve soil P acquisition by plants[13]. Adaptation of crop varieties and/or crop rotations to include P-mobilising species has large potential to improve P acquisition in tropical soils with large reserves of non-labile P and limited reserves of plant available P. Exudation of carboxylates, modulation of acid and/or alkaline phosphatase activity, root morphology acclimation and proton release to decrease rhizosphere pH are mechanisms employed by plants adapting to low P availability[14]. For example, in P-limiting conditions, sugarcane varieties can show variable adaptations to increase P uptake based on their root biomass[15]. Merlin et al.[16] recently showed that Brachiaria (*Brachiaria ruziziensis*) grown as a cover crop can take up P bound to Al and Fe oxides in tropical acidic soils and potentially make it more available to succeeding crops. Sousa et al.[17] showed that the critical P level for soybean in an integrated crop-livestock system was half that needed in an annual cropping system. Pigeon pea increased P uptake of intercropped sorghum by exuding piscidic acid that chelates Fe, and subsequently releases P from iron phosphate ($FePO_4$)[18].

Microbial engineering: Several groups of bacteria including the genera *Rhizobium*, *Enterobacter, Agrobacterium*, *Azotobacter* and *Erwinia*, and fungi affiliated to the genus *Aspergillus and Penicillium* have shown a capacity to mobilise soil organic and inorganic P from tropical soils via the release of enzymes (phytases and phosphatases), protons and

organic acids (gluconic, citric, oxalic, succinic or tartaric), most notably in laboratory-based experiments[19,20]. Uptake of mobilised P into microbial biomass can then be actively recycled to provide soluble P for plant uptake. For example, Mirza et al.[21] attributed up to 55% of in-vitro sugarcane growth to the P availability promoted by bacteria affiliated to the genus *Enterobacter*. Mycorrhizal fungi also play an important role in soil P acquisition through hyphal extension of plant roots[22].

Although the mechanisms of P mobilization by microorganisms are well known, microbial activity in field soils cannot currently be relied upon to sustain P supply for crops because of limited understanding of the microbial ecology of P-mobilizing genera. There are no data showing how abundant, or how diverse, the microbial communities involved in P cycling should be. Optimising P supply may require a large diversity of soil microbes and/or communities that exhibit functional redundancy[23], and microbial engineering offers the potential to prescribe microbial recipes for specific cropping systems, cultivation regimes, soil types and climatic regions. One can hypothesize that the greater the microbial diversity, the greater the microbial activity and the greater the likelihood of mobilizing moderately-labile or non-labile P in tropical soils[24,25]. Research is only just beginning to explore how engineering of the soil microbiome can support the utilization of legacy soil P.

Fertilizer engineering: Novel P fertilizers developed through various bio-technologies, and or P recovery strategies, have been evaluated, or used commercially in Brazil in order to increase the efficiency of P fertilizers [26]. Key to the success of these technologies is producing fertilizers with a low water P solubility, and a slow pattern of P release to more accurately match crop P demand and reduce susceptibility to rapid immobilization (adsorption and precipitation) of P by Fe and Al oxides in the soil. For example, struvite recovered from wastewater, or from livestock manures, has been shown to provide a slow-release and efficient P supply to crop without sacrificing productivity[27,28]. However, it is noteworthy that while many novel fertilizers have been advocated for use on farms by the fertilizer industry in Brazil, there remains little scientific validation of improvements in P use efficiency in the field. Improved targeting of P through a better understanding of physiological demand through the growing season, and more innovative application technologies via seed

dressings, placement and foliar applications may further enhance the role of precision farming in the P sustainability of Brazilian crop production systems[29].

References

1. IBGE. Estatísticas Resultados de pesquisas. Instituto Brasileiro de Geografia e Estatística. https://sidra.ibge.gov.br (22 November 2017) (2017).
2. Sparovek, G., Barretto, A. G. D. O. P., Matsumoto, M. & Berndes, G. Effects of governance on availability of land for agriculture and conservation in Brazil. Environ. Sci. Technol. 49(17), 10285-10293 (2015).
3. Franchini, J. C. et al. Evolution of crop yields in different tillage and cropping systems over two decades in southern Brazil. F. Crop. Res. 137, 178–185 (2012).
4. Lopes, A. S. S. & Guilherme, L. R. G. A career perspective on soil management in the Cerrado region of Brazil. Adv. Agron. 137, 1–72 (2016).
5 Strassburg, B. B. N. et al. When enough should be enough: Improving the use of current agricultural lands could meet production demands and spare natural habitats in Brazil. Glob. Environ. Change 28, 84–97 (2014).
6. Gatiboni, L. C., Kaminski, J., Rheinheimer, D. S. & Flores, J. P. C. Bioavailability of soil phosphorus forms in no-tillage system. R. Bras. Ci. Solo, 31, 691-699 (2007).
7. Gatiboni, L. C. Availability of Phosphorus Forms to Plants [Ph.D. thesis]. Federal University of Santa Maria, 231 p (2003).
8. Gatiboni, L. C., Kaminski, J., Rheinheimer, D. S. & Brunetto, G. Soil microbial biomass phosphorus and activity of acid phosphatases during decline of soil available phosphorus. Pesq. Agropec. Bras., 43, 1085-1091 (2008).
9. Raboy, V. Approaches and challenges to engineering seed phytate and total phosphorus. Plant Sci. 177, 281–296 (2009).
10. Wang, F., Rose, T., Jeong, K., Kretzschmar, T. & Wissuwa, M. The knowns and unknowns of phosphorus loading into grains, and implications for phosphorus efficiency in cropping systems. J. Exp. Bot. 67, 1221–1229 (2016).
11. ANDA. Estatísticas Indicadores: Principais Indicadores do Sector de Fertilizantes. http://anda.org.br/index.php?mpg=03.00.00 (23 November 2017) (2017).
12. Prado, R. de M., Caione, G. & Campos, C. N. S. Filter Cake and Vinasse as fertilizers contributing to conservation agriculture. Appl. Environ. Soil Sci., 581984, 8 pp (2013).
13. Wang, X., Shen, J. & Liao, H. Acquisition or utilization, which is more critical for enhancing phosphorus efficiency in modern crops? Plant Sci. 179, 302–306 (2010).
14. Lambers, H., Martinoia, E. & Renton, M. Plant adaptations to severely phosphorus-impoverished soils. Curr. Opin. Plant Biol. 25, 23–31 (2015).
15. Arruda, B. et al. Biological and morphological traits of sugarcane roots in relation to phosphorus uptake. J. Soil Sci. Plant Nutr. 16(4), 901-905 (2016).
16. Merlin, A., Rosolem, C. A. & He, Z. Non-labile phosphorus acquisition by Brachiaria. J. Plant Nutr. 39, 1319–1327 (2016).
17. Sousa, D. M. G. de., Nunes, R. S., Rein, T. A. & Santos Junior, J. D. G. dos, Manejo do fósforo na região do Cerrado. In: Flores, R. A.; Cunha, P. P. da (eds.). Práticas de manejo do

solo para adequada nutrição de plantas no Cerrado, 1. ed. Goiânia: Gráfica UFG, p. 291-358 (2016).
18. Ae, N., Arihara, J., Okada, K., Yoshihara, T. & Johansen, C. Phosphorus uptake by Pigeon Pea and its role in cropping systems of the Indian subcontinent. Science 248(4954), 477-480 (1990).
19. Azziz, G. et al. Abundance, diversity and prospecting of culturable phosphate solubilizing bacteria on soils under crop–pasture rotations in a no-tillage regime in Uruguay. Appl. Soil Ecol. 61, 320–326 (2012).
20. Kaur, G. & Reddy, M. S. Effects of phosphate-solubilizing bacteria, rock phosphate and chemical fertilizers on maize-wheat cropping cycle and economics. Pedosphere 25, 428–437 (2015).
21. Mirza, M. et al. Isolation, partial characterization, and the effect of plant growth-promoting bacteria (PGPB) on micro-propagated sugarcane in vitro. Plant Soil 237, 47–54 (2001).
22. Chagnon, P.-L., Bradley, R. L., Maherali, H. & Klironomos, J. N. A trait-based framework to understand life history of mycorrhizal fungi. Trends Plant Sci. 18, 484–491 (2013).
23. Allison, S. D. & Martiny, J. B.H. Resistance, resilience, and redundancy in microbial communities. Proc. Natl. Acad. Sci. USA 105: 11512-11519 (2008).
24. Rodrigues, J. L. M. et al. Conversion of the Amazon rainforest to agriculture results in biotic homogenization of soil bacterial communities. Proc. Natl. Acad. Sci. 110, 988–93 (2013).
25. Paula, F. S. et al. Land use change alters functional gene diversity, composition and abundance in Amazon forest soil microbial communities. Mol. Ecol. 23, 2988–2999 (2014).
26. Benites, V. de M. A importância da pesquisa na avaliação da eficiência das tecnologias em fertilizantes fosfatados no Brasil. Boletim Informativo da SBCS 40(2): 36-38, (2015).
27. Talboys, P. J., Heppell, J., Roose, T., Healey, J. R., Withers, P. J. A., Jones, D. L. Struvite: a slow-release fertilizer for sustainable phosphorus management? Plant Soil 401:109–123 (2016).
28. Achat, D. L., Sperandio, M., Daumer, M. L., Santellani, A. C., Prud'Homme, L., Akhtar, M. & Morel, C. Plant availability of phosphorus recycled from pig manures and dairy effluents as assessed by isotopic labeling techniques. Geoderma 232-234, 24–33 (2014).
29. Sylvester-Bradley, R. et al. Improving the sustainability of phosphorus use in arable farming – ‘Targeted P’. AHDB Project Report No. 569, AHDB, Stoneleigh, UK. https://cereals.ahdb.org.uk/publications/2017/june/12/improving-the-sustainability-of-phosphorus-use-in-arable-farming-%E2%80%93-%E2%80%98targeted-p%E2%80%99.aspx (27 November 2017) (2017).
